# P18 - Contact sensitisation in children and adolescents: the experience of the University Hospital (Hospital de Clínicas) in Uruguay

**DOI:** 10.1186/2045-7022-4-S1-P73

**Published:** 2014-02-28

**Authors:** Iris S Ale, Valeria Pomiés, Patricia Levrero

**Affiliations:** 1 Department of Allergology and Department of Dermatology, Republic University of Uruguay, Montevideo, Uruguay

## Background

Allergic contact dermatitis (ACD) is often under-recognized in the pediatric population. In the first years of life ACD it is often confused with other types of dermatitis, such as irritant dermatitis or atopic eczema (AE). In the last two decades an increase in ACD in children and adolescents was observed, reaching similar frequencies to those seen in adults (20-70%). ACD acquired in childhood may have a significant impact for patients and their families, and may compromise decisions concerning occupation in adulthood.

## Methods

We conducted a retrospective review including all children and adolescents 18 years or younger with a suspected ACD, attending the Unit of Allergy and Department of Dermatology, University of Uruguay, over a 9-year period (January 1, 2004–June 30, 2013). Patch tested was performed with allergens from the International Standard Series and additional allergens reported to be frequent in children and others which were relevant to the clinical situation.

## Results

One-hundred fifty-seven children and adolescents (61% females and 39% males) were patch tested. Twenty-three percent of them were 10 years or younger. Fifty-nine percent of the children and 53 percent of the adolescents tested positive to at least one allergen. Sixty percent of these reactions were deemed to be of current relevance, 16% have probable or possible relevance, 11% past relevance and 13% of all positive reactions were of unknown relevance. The most common allergens were nickel, neomycin, thimerosal, fragrance mix, cobalt and thiuram mix. Very few irritant reactions were seen and no active sensitization was observed.

## Conclusion

Contact sensitization is an important pathogenic factor for the development of dermatitis in children and adolescents. Patch testing should be utilized more frequently as a valuable diagnostic tool in the pediatric setting when the clinical presentation is suggestive of ACD.

